# Immune Checkpoint Therapy for Thymic Carcinoma

**DOI:** 10.3390/cancers17203377

**Published:** 2025-10-20

**Authors:** Jinhui Li, Fuling Mao, Hongyu Liu, Jun Chen

**Affiliations:** 1 Department of Lung Cancer Surgery, Tianjin Medical University General Hospital, Tianjin 300052, China; lijinhui2025@foxmail.com (J.L.); maofuling@tmu.edu.cn (F.M.); 2 Tianjin Key Laboratory of Lung Cancer Metastasis and Tumor Microenvironment, Tianjin Lung Cancer Institute, Tianjin Medical University General Hospital, Tianjin 300052, China

**Keywords:** thymic carcinoma, immune checkpoints inhibitors, programmed cell death protein ligand 1, programmed cell death protein 1, immune-related adverse events

## Abstract

**Simple Summary:**

Thymic carcinoma (TC) is rare and aggressive. For unresectable or advanced disease, platinum-based chemotherapy remains first-line, but a durable benefit is uncommon. PD-1/PD-L1 inhibitors provide an option after platinum in selected patients: objective responses occur in a minority, disease control in more, and signals are stronger in PD-L1–positive tumors despite low tumor mutational burden. Toxicities appear less frequent than in thymoma, yet hepatitis, myositis, and myocarditis require vigilant monitoring. Beyond monotherapy, combinations with anti-angiogenic agents or chemotherapy are being tested and may extend control in subsets. This review synthesizes efficacy, safety, and biomarkers of immune checkpoint blockade in TC, and offers practical points on perioperative use, patient selection, and surveillance.

**Abstract:**

Thymic carcinoma (TC) is a rare, aggressive cancer that originates from thymus’s epithelial cells. It distinguishes itself from other thymic epithelial tumors with its unique pathological structure, clinical behavior, and immune characteristics. Immune checkpoint inhibitors (ICIs) targeting the Programmed cell death protein 1/Programmed cell death protein ligand 1 (PD-1/PD-L1) pathway have shown promise in advanced TC, potentially benefiting from frequent PD-L1 overexpression and abundant CD8^+^ tumor-infiltrating lymphocytes (TILs), despite typically low tumor mutational burden (TMB). While ICI monotherapy can achieve disease control in some patients, its overall efficacy is limited and it is associated with a distinct profile of immune-related adverse events (irAEs) which occur less often than in thymomas. The predictive value of biomarkers—particularly PD-L1 expression—remains uncertain, underscoring the importance of consistent assessment criteria. In this review, we summarize evidence on ICI monotherapy as well as combination approaches that incorporate anti-angiogenic agents, chemotherapy, or dual checkpoint blockade. Emerging therapeutic targets—such as CD70, TIM-3, and B7-H4—are also considered in the context of their potential clinical relevance. Finally, we discuss future directions aimed at improving efficacy, extending response durability, and reducing treatment-related toxicity through biomarker-based patient selection and tailored therapeutic strategies.

## 1. Introduction

Thymic epithelial tumors (TETs) are rare malignant tumors originating from the thymic epithelium that include TC, thymomas, and thymic neuroendocrine tumors (NETTs) [1]. Unlike thymoma, TC is typically diagnosed at an advanced stage and is associated with poor long-term survival [2]. Compared with thymoma, TC displays more cytological atypia, invasive behavior, and poor prognosis. Due to its rarity and frequent late-stage diagnosis, systemic therapy is the cornerstone for unresectable TC.

For many years, platinum-based chemotherapy—commonly in the form of multi-agent regimens such as ADOC (cisplatin, doxorubicin, vincristine, and cyclophosphamide) or CAP (cisplatin, doxorubicin, and cyclophosphamide)—has served as the standard first-line approach for TC. These regimens typically yield objective response rates (ORRs) of 40–50% [3], but responses are often transient, with median Progression-Free Survival (mPFS) limited to 5–8 months [4]. Second-line agents, including S-1, amrubicin, and pemetrexed, generally produce ORRs below 20% [5,6,7], and no consensus exists for treatment beyond first-line therapy. In addition, the high rate of disease recurrence underscores the need for more effective and durable systemic options.

TC frequently shows PD-L1 expression and abundant CD8^+^ TILs, supporting evaluation of PD-1/PD-L1 blockade despite generally low TMB [8,9,10]. Early studies report ORRs around 19–23% and durable disease control in a subset [10,11]. Nonetheless, irAEs—including myocarditis, myositis, and hepatitis—can be serious, highlighting the importance of careful patient selection. The predictive value of biomarkers such as PD-L1 and PD-1 remains inconsistent, owing in part to methodological variability and biological heterogeneity.

Surgical resection remains the mainstay of treatment for TETs, particularly TC, with R0 resection linked to improved survival. However, 40–60% of TC cases present with locally advanced disease at diagnosis, often precluding upfront surgery [12]. Even after resection, TC shows high 10-year recurrence rates (59–76%), significantly higher than thymoma (29–71%) [13]. These challenges have spurred growing interest in integrating ICIs into perioperative management. This review will examine the current evidence regarding the efficacy, safety, and biomarkers for ICIs treatment, with the aim of exploring future strategies and improving clinical outcomes for TC patients.

## 2. Immunological Landscape of Thymic Carcinoma

Across immunohistochemical studies using different clones (e.g., 22C3, 28-8, SP142, SP263) and positivity definitions (e.g., TPS and H-score thresholds), the prevalence in TC ranges from approximately 27% to 92.5% [14,15,16] (Table 1). In a comparative cohort, Katsuya et al. observed PD-L1 positivity in 70% of TC versus 23% of thymoma cases [9]. The relationship between PD-L1 expression and clinicopathological features is inconsistent.

Several studies reported no significant associations with age, sex, tumor stage, histologic subtype, tumor size, or resectability [9,16,17]. Studies, such as Funaki et al. [18] observed increased PD-L1 expression following chemotherapy, raising the possibility of treatment-induced upregulation.

The prognostic value of PD-L1 expression in TC remains inconclusive. Some studies have reported an association between high PD-L1 expression and improved survival outcomes [19,20], while others have found a negative correlation or no significant relationship [18,21]. For instance, Yokoyama et al. observed better overall and disease-free survival in patients with high PD-L1 expression [19], whereas Padda et al. reported worse survival after adjusting for age and sex [22]. Conversely, several studies, including those by Higuchi et al. [23] and Rouquette et al. [16], did not demonstrate a clear association. These discrepancies may be attributed to variations in study populations, sample sizes, disease stages, antibody clones, scoring systems, and cut-off thresholds. In addition, the prognostic role of PD-1 expression and its interaction with PD-L1 has not been well characterized. Overall, current data does not support a consistent prognostic effect of PD-L1 in TC, and routine use of PD-L1 for risk stratification remains premature without assay harmonization and prospective validation.

**Table 1:** PD-L1 Expression and Survival Associations in TC.

Year	Author	N	PD-L1 Antibody	Cut-Off	PD-L1^+^ (%)	Survival Association (High vs. Low)
2018	Chen et al. [24]	20	SP142	≥3%, (% × intensity)	70%	NS
2018	Sakane et al. [20]	53	SP142, SP263, 22C3, 28-8	≥1%/25%, (%)	49.1~92.5%	Longer OS
2018	Funaki et al. [18]	43	SP142	≥50%, (TPS)	60.5%	Worse prognosis
2018	Duan et al. [21]	20	ab58810	median, (% × intensity)	65%	Shorter mPFS
2019	Song et al. [17]	60	SP263	≥50%, (TPS)	35%	NS
2019	Higuchi et al. [23]	6	28-8	≥1%, (TPS)	63%	NS
2019	Rouquette et al. [16]	50	E1L3N, SP142, 22C3, SP263	≥1%/50%, H-score	20~24% (50%) 66~73% (1%)	NS
2020	Bedekovics et al. [25]	7	SP142	TC ≥ 50% or IC ≥ 10%, (%)	43%	NS
2020	Beradi et al. [26]	4	28-8	≥1%, (TPS)	50%	NS
2020	Ishihara et al. [14]	11	SP263	≥25%, (TPS)	27%	Shorter RFS
2021	Wang et al. [15]	15	22C3 or 28-8	≥1%, (%)	54.6%	NS
2022	Kashima et al. [27]	31	E1L3N	≥1%, (%)	74%	NS
2025	Chubachi et al. [28]	11	SP142, SP263	≥1%, (%)	73~82%	NS
2025	Lu et al. [29]	34	SP142	≥1%, (%)	41.7%	Longer PFS

% × intensity, product of positive cell percentage and the staining intensity; NS, not significant; PD-L1, programmed death ligand 1; TC, Thymic carcinoma; TPS, Tumor Proportion Score; mPFS, median Progression-Free Survival; RFS, Recurrence-free survival.

Despite these uncertainties, other immune features of TC suggest potential sensitivity to immune checkpoint blockade. It was shown that all TET samples exhibited high levels of CD3^+^ and CD8^+^ TIL infiltration, with CD8^+^ lymphocytes accounting for approximately 82.8 ± 18.3% of CD3^+^ lymphocytes [23,30]. In addition, 62% of patients with TC showed high PD-1 expression on TIL, suggesting the presence of an immunosuppressive microenvironment that could potentially be reversed by ICI therapy [23]. Although TC generally exhibits low TMB and microsatellite stability (MSS), responses to ICIs have been reported even in TMB-low cases [30,31]. For example, in a phase II trial, Giaccone et al. [10] documented durable responses to pembrolizumab in patients with MSS and low TMB, suggesting that factors such as TIL density or PD-L1 expression may serve as more meaningful predictors of benefit than TMB alone. Building upon T-cell-centered strategies, growing evidence suggests that natural killer (NK) cells also contribute to tumor immunity and represent promising therapeutic targets in TC.

Compared to the extensively studied T-cell-mediated immune responses, the role of NK cells in TETs remains insufficiently elucidated. Histological analyses have revealed that NK cell infiltration is generally limited in TC, suggesting the presence of an immune-excluded or immunosuppressive tumor microenvironment. This phenomenon may be influenced by the expression of immune checkpoint molecules which are believed to attenuate NK cell activity and facilitate tumor immune evasion. In clinical studies of avelumab, an anti–PD-L1 antibody, patients who achieved favorable responses exhibited lower baseline levels of peripheral NK cells and B cells, along with greater T-cell receptor (TCR) diversity, a feature associated with enhanced immune responsiveness [32]. Additionally, patients who developed irAEs were found to have higher baseline levels of CD4^+^ T cells and reduced NK-T-cells, indicating a potential link between these immune subsets and toxicity mechanisms [33]. Notably, these immunologic correlations were derived largely from thymoma cohorts or mixed TET populations rather than TC-specific series and should be interpreted as exploratory signals pending TC-focused validation.

Beyond these mechanistic insights, novel therapeutic strategies have emerged to potentiate NK cell function as a means of augmenting immune-based treatments. For example, nanrilkefusp alfa—an IL-15 receptor agonist—has demonstrated the ability to promote proliferation and activation of NK and CD8^+^ T-cells without significantly expanding regulatory T-cells (Tregs) [34,35]. Preliminary findings suggest that this agent can induce disease stabilization in patients with advanced TETs, and no additional toxicity was observed when combined with PD-1 inhibitors. Another agent, PT-112, induces immunogenic cell death and has shown the capacity to activate multiple immune subsets, including NK cells [36]. In a recent phase II clinical trial targeting relapsed TETs, PT-112 achieved a high rate of disease control and was associated with increases in circulating CD8^+^ T-cells, activated CD4^+^ T-cells, and NK cells [37]. These findings collectively indicate that NK cell-targeted therapies may serve as a valuable complement to existing T-cell-oriented immunotherapeutic approaches.

In summary, although PD-1 and PD-L1 are expressed in TC and may contribute to immune escape, their clinical relevance as predictive biomarkers remains uncertain. Variable findings across studies are likely due to methodological differences, including antibody clones and scoring systems. Until standardized assays and prospective validation studies are conducted, the routine use of PD-L1 for patient selection remains premature.

## 3. Clinical Trials of ICI Monotherapy for TC Patients

The exploration of ICIs in TC has gained momentum in recent years, largely due to the limited systemic treatment options available for metastatic disease following platinum-based chemotherapy. Given the typically aggressive course and poor prognosis of metastatic TC, ICIs have been introduced as a rational therapeutic option. However, their clinical use remains complicated by a significant risk of irAEs, requiring careful patient evaluation and close monitoring. Several phase II studies have therefore assessed ICI monotherapy in metastatic or advanced cases, though outcomes have varied depending on the specific agents used and the characteristics of the treated populations (Table 2).

### 3.1. Pembrolizumab

In a phase II trial conducted by Giaccone et al. [10], pembrolizumab was administered to 40 patients with previously treated TC. The ORR was 22.5%, and the disease control rate (DCR) reached 75%. mPFS was 4.2 months, while median overall survival (mOS) extended to 24.9 months. Notably, patients exhibiting high PD-L1 expression demonstrated significantly prolonged PFS and OS compared to those with low or absent expression, suggesting a potential predictive role of this biomarker. A genomic analysis from the same cohort further identified alterations in the CYLD and BAP1 genes as possible molecular correlates of PD-L1 status and ICI responsiveness [38].

Similar efficacy outcomes were observed in an independent Korean phase II study by Cho et al. [11], which enrolled 26 patients with refractory TC. In this cohort, pembrolizumab achieved an ORR of 19.2% and a DCR of 73.1%. Median PFS and OS were reported at 6.1 and 14.5 months, respectively. Taken together, these findings support the durable clinical activity of pembrolizumab in a subset of patients with TC and underscore the importance of identifying robust predictive biomarkers to guide patient selection and optimize therapeutic benefit.

**Table 2:** Clinical Outcomes of ICI Monotherapy in TC.

Treatment	Pathological Type	Number of Patients	Median Age/y	Male/ Female	ORR (%)	DCR (%)	mPFS (Months)	mOS (Months)	Primary endpoint	Rate of Severe irAEs (%)	Reference
Pembrolizumab	TC	40	57	28/12	22.5	75	4.2	24.9	ORR	15	Giaccone et al., 2018 [10]
Pembrolizumab	TC	26	57	18/8	19.2	73.1	6.1	14.5	ORR	15.4	Cho et al., 2019 [11]
Nivolumab	TC	15	55	12/3	0	73.3	3.8	14.1	ORR	14.3	Katsuya Y., 2019 [39]
Nivolumab	T and TC	10/43	58	35/20	12	67	6.2	21.3	PFS	59	N. Girard., 2023 [40]
Atezolizumab	TC	34	55	26/8	14.7	58.8	3.2	NA	ORR	5.9	Lu et al., 2025 [29]

TC, Thymic carcinoma; T, Thymoma; ORR, Overall Response Rate; DCR, Disease Control Rate; mPFS, median Progression-Free Survival; mOS, median Overall Survival; irAEs, immune-related Adverse Events.

### 3.2. Nivolumab

The PRIMER study [39] evaluated nivolumab in 15 patients with previously treated TC. While the DCR was 73%, no objective responses were observed, and median PFS and OS were 3.8 and 14.1 months, respectively. The study was discontinued early due to insufficient efficacy. In contrast, the international NIVOTHYM trial [40] enrolled 43 patients with TC and achieved a modest ORR of 12% and DCR of 63%, with a 6-month PFS of 6.2 months. These mixed results suggest that nivolumab may offer limited benefit in TC monotherapy, especially compared to pembrolizumab.

A small retrospective study [41] of low-dose nivolumab (due to toxicity concerns) also showed limited efficacy, with only partial responses in a few patients and a median OS of 7.4 months. Notably, severe irAEs were common, further complicating its usage.

### 3.3. Atezolizumab

Atezolizumab, a PD-L1 inhibitor, has been evaluated in two prospective studies. A multinational basket trial (NCT02458638) [42] enrolled 12 patients with advanced TC, reporting an 18-week non-progression rate (NPR) of 41.7% and an ORR of 8.3%. These modest outcomes suggest limited single-agent activity in this population.

More recently, a multicenter, single-arm phase II study was conducted in China [29] enrolled 34 patients with advanced TC refractory to prior therapies. The confirmed ORR was 14.7%, with a DCR of 58.8%, median PFS of 3.2 months, and a 24-month OS rate of 63.2%. Importantly, patients with PD-L1 expression ≥ 1% on tumor or immune cells showed significantly improved outcomes, with an ORR of 40.0% and median PFS of 7.4 months, compared to PD-L1–negative patients.

Overall, PD-1/PD-L1 monotherapy in previously treated TC shows limited activity, with low double-digit response rates and short mPFS. Outcomes appear enriched in tumors with PD-L1 ≥ 1%, supporting post-platinum use in selected patients; however, the lack of validated cut-offs precludes routine biomarker-guided selection. Given the risk of toxicities, monotherapy is most defensible when close surveillance is feasible and subsequent options (e.g., anti-angiogenic combinations or chemotherapy doublets) are preplanned. Randomized trials are required to determine whether combination regimens can consistently outperform single-agent therapy [43].

## 4. Immune-Related Adverse Events (irAEs) in Thymic Carcinoma

### 4.1. Overview of irAE Profile in Thymic Carcinoma

**Figure 1:**
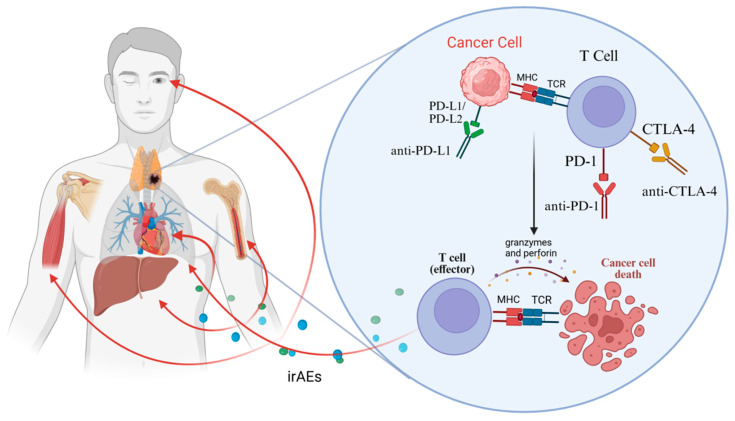
Mechanism of anti-PD-1/PD-L1 and CTLA-4 antibodies. Color/shape key: cancer cell (pink); T cell (purple); PD-L1/PD-L2 (green ligands on the cancer cell); PD-1 (red receptor on T cell); CTLA-4 (gold receptor on T cell); MHC (red peptide–MHC complex on the cancer cell); TCR (blue receptor on T cell); therapeutic antibodies (Y-shaped icons: anti-PD-1, anti-PD-L1, anti-CTLA-4). Arrows: black solid arrow = T-cell activation leading to granzyme/perforin release and cancer-cell death; red solid arrows toward organs = potential sites of irAEs. Dotted particles represent cytotoxic mediators released by activated T cells. Abbreviations/definitions: PD-L2, programmed death-ligand 2 (second ligand for PD-1, expressed on antigen-presenting cells and some tumors); MHC, major histocompatibility complex presenting peptide antigens to T cells; TCR, T-cell receptor recognizing peptide–MHC to initiate activation; irAEs, immune-related adverse events caused by off-target immune activation during checkpoint blockade.

As illustrated in Figure 1, TC exhibits a distinct immune-related toxicity profile compared with thymomas. The incidence of grade ≥ 3 irAEs in TC is approximately 15%, markedly lower than in thymomas (up to 71%) [11]. Severe toxicities most often involve hepatitis (elevated ALT/AST), myositis, and occasionally myocarditis or ocular symptoms [10] (Table 3). Interestingly, several reports have described higher partial remission rates among patients who developed irAEs [10], raising the possibility that immune activation and treatment efficacy may be linked. Such patterns may be rooted in the unique immunobiology of the thymus, but differences from thymoma—especially the high-risk B3 subtype—merit closer examination.

### 4.2. Incidence and Clinical Spectrum

While B3 thymoma highlights an extreme example of immune toxicity risk, the broader profile in TC is one of lower incidence but meaningful clinical impact. Across clinical trials of ICIs, grade ≥ 3 irAEs occur in roughly 10–20% of TC patients, far below the levels reported in thymomas. Severe toxicities are generally manageable with corticosteroids or other immunosuppressants, but early recognition remains essential. Notably, in the phase II trial by Giaccone et al. [10], four of nine patients who experienced severe irAEs achieved partial remission, supporting the idea that toxicity may correlate with anti-tumor activity.

Observational data from VigiBase further reinforce the elevated reporting rates of ICI-induced cardiomyopathy (rOR 17.9, 95% CI: 8.9–23.4) and myositis (rOR 14.4, 95% CI: 8.9–23.4) in TETs relative to other malignancies [44]. In a Japanese study by Katsuya et al. [39], nivolumab-treated TC patients mostly developed mild events, with a single case of grade 3 AST elevation. By contrast, a Chinese phase II trial of atezolizumab [29] documented an overall irAE incidence of 29.4%, but only 5.9% grade ≥ 3 events and no grade 4–5 toxicities, suggesting a comparatively favorable safety profile.

### 4.3. Contraindications and Special Populations

Outside clinical trials, patients with moderate-to-severe, active autoimmune disease—particularly with neuromuscular or cardiac involvement—generally do not receive ICIs because of the risk of disabling myositis or myocarditis [8]. Relative contraindications include refractory autoimmune thyroiditis with myopathy, a history of ICI-associated myocarditis, or a recent need for intensified systemic immunosuppression [45]. Although high-grade irAEs appear less frequent in TC than in thymoma, decisions should remain conservative. Before starting treatment, undertake a focused immune and cardiac work-up: request ANA and organ-specific autoantibodies (e.g., AChR) when clinically indicated; obtain baseline CK and thyroid function; and in symptomatic patients or those with cardiac risk factors, perform an ECG with high-sensitivity troponin [46]. Patients receiving chronic corticosteroids or other immunosuppressants should be counseled about potentially reduced efficacy and altered toxicity kinetics.

During early cycles, review symptoms and check laboratories (CK, AST/ALT, thyroid tests) at regular intervals, and keep a low threshold for cardiology/neurology input if myalgia, weakness, chest pain, or dyspnea develops. Suspected grade ≥ 2 myositis or myocarditis warrants immediate treatment hold, prompt corticosteroids according to guidance, and multidisciplinary oversight; any re-challenge should be individualized and deferred until complete clinical and biochemical resolution with discontinuation of steroids or only physiologic dosing [44]. Because most ICI trials exclude patients with active or clinically significant autoimmune disease, eligibility thresholds and monitoring intensity in TC cannot simply be imported from other cancers [40]. TC-specific registries and prospective studies are needed to refine selection criteria and to identify markers that predict severe irAEs.

In summary, although TC carries a substantially lower risk of high-grade irAEs than thymoma, its toxicity spectrum warrants careful monitoring. Baseline assessments, routine laboratory surveillance, and coordinated multidisciplinary care are critical to reducing morbidity. Future work should define ICI-specific toxicity signatures in TC and refine strategies for risk-based patient selection.

## 5. Novel ICIs and Targeted Therapies for Thymic Carcinoma

### 5.1. Next-Generation Immune Targets

CD70 is aberrantly expressed in several malignancies, including TC, while its expression is limited in normal tissues [27]. Its interaction with CD27 promotes T-cell activation but also contributes to T-cell exhaustion and immune evasion in tumors [47,48]. Preclinical studies have demonstrated that CD70-targeted agents, such as antibody–drug conjugates (ADCs) and CAR-T-cells, exhibit anti-tumor activity in CD70-positive tumors. A phase I trial of CTX130, an allogeneic CD70-directed CAR-T therapy, demonstrated a 46.2% ORR in relapsed or refractory T-cell lymphoma, highlighting its translational potential in CD70-positive tumors [49].

TIM-3 (HAVCR2), an inhibitory receptor expressed on T-cells, negatively regulates Th1 responses and contributes to immune evasion [50]. Its blockade enhances interferon-γ secretion and may synergize with PD-1/PD-L1 or CTLA-4 inhibitors [51]. Moderate to high TIM-3 expression has been observed in most TC, despite limited correlation with PD-L1, supporting its potential as a combinatorial immunotherapy target. [52]

B7-H4 (VTCN1), a member of the B7 family, inhibits T-cell activation and is overexpressed in various malignancies, including TETs [53]. In a comprehensive study, Wang et al. [54] reported B7-H4 expression in approximately 79% of TET cases, with significant associations to clinicopathologic features. Notably, co-expression of B7-H4 and PD-L1 may correlate with disease progression, supporting the rationale for dual immune checkpoint blockade in TC.

### 5.2. Non-T-Cell Immunoregulators

Indoleamine-2,3-dioxygenase (IDO) is an intracellular enzyme that catabolizes tryptophan into kynurenine, depleting this essential amino acid and generating immunosuppressive metabolites [55]. This process impairs T-cell function and facilitates immune escape. In TETs, IDO is often overexpressed, and lower expression levels have been associated with improved survival. These findings suggest that IDO inhibition may represent a viable therapeutic strategy in TC [56].

Transforming growth factor-beta (TGF-β) is a pleiotropic cytokine involved in tissue remodeling and immune regulation [57,58]. Overexpression of TGF-β in TC contributes to epithelial–mesenchymal transition, immune evasion, and suppression of cytotoxic CD8+ T-cell proliferation. High TGF-β levels have been linked to poorer overall survival, reinforcing its potential as a therapeutic target in advanced TC [57,59].

### 5.3. Emerging Therapeutic Targets

Calmodulin-like protein 5 (CALML5) has recently emerged as a novel target in TC [60]. Overexpression of CALML5 promotes cell proliferation and enhances cisplatin sensitivity in preclinical models [61]. Gene set enrichment analysis indicated upregulation of E2F-related transcription pathways, supporting its potential role in TC progression.

Mucin 1 (MUC1) is highly expressed in TC and correlates with tumor aggressiveness and poor prognosis. MUC1 expression was significantly higher in TC than in B3 thymomas (94% vs. 0%), with a sensitivity of 94%, and may be a useful marker for differentiating TC from B3 thymomas [62]. MUC1 is also involved in oncogenic signaling and immune evasion. Vaccine-based therapies targeting MUC1 are under development, highlighting its translational promise [63,64].

SRY-box transcription factor 9 (SOX9) is implicated in the maintenance of stemness and induction of epithelial–mesenchymal transition [65]. High SOX9 expression in TC is associated with increased invasiveness and worse prognosis [66,67]. Bioinformatics analyses have confirmed its correlation with unfavorable gene expression profiles, suggesting its role as both a prognostic marker and therapeutic target [68].

Trophoblast cell surface antigen 2 (Trop-2), a membrane glycoprotein involved in cell signaling and proliferation, is strongly expressed in TC but not in normal thymic tissue [69]. Trop-2 overexpression is linked to tumor progression in various malignancies, and several Trop-2-targeted therapies are under clinical investigation. Its expression profile supports its candidacy as a therapeutic target in TC [70].

## 6. Combination Therapies for Thymic Carcinoma

As research in this area intensifies, new combination strategies are being explored to improve the efficacy of ICIs in TC. These approaches include combining ICIs with anti-angiogenic drugs, chemotherapy and dual immune checkpoint blockade [8] (Table 4).

### 6.1. Combination Therapy with ICIs and Anti-Angiogenic Drugs

Vascular endothelial growth factor promotes immune evasion by inhibiting T-cell function and dendritic cell maturation. It promotes the development of an immunosuppressive tumor microenvironment by increasing intertumoral Tregs and myeloid-derived suppressor cells [71]. Combining ICIs with anti-angiogenic drugs has shown promise, especially in patients who have not previously received anti-angiogenic therapy [72].

CAVEATT [73] was a single-arm, multicenter, phase II trial, conducted by Conforti et al., that evaluated the efficacy of avelumab in combination with the anti-angiogenic drug axitinib in the treatment of advanced B3 thymoma and TC patients. Twenty-seven of the 32 participants were patients with histologically confirmed TC who had progressed after platinum-based chemotherapy. The overall response rate was 34%, with median PFS and median OS of 7.5 and 26.6 months, respectively; disease control was achieved in more than 90% of patients, which was better than that with avelumab monotherapy. These results, derived predominantly from TC, suggest that PD-L1 blockade may require concurrent VEGFR inhibition to achieve clinically meaningful depth and durability of response in this histology, aligning with the limited signal seen with PD-L1 monotherapy.

### 6.2. Combination Therapy with ICIs and Chemotherapy

Recent studies have explored the efficacy of combining pembrolizumab with chemotherapy in the treatment of metastatic TC. A case report [74] described two patients with metastatic TC who achieved durable clinical responses to first-line pembrolizumab combination chemotherapy. One patient achieved complete remission for more than three years and the other maintained partial remission for more than 20 months. This suggests that combining pembrolizumab with chemotherapy may be an effective strategy for the first-line treatment of metastatic TC, even for patients with a low TMB and MSS. In addition, the Marble [75] study, a multicenter, single-arm, open-label, phase II trial investigated the safety and efficacy of atezolizumab in combination with carboplatin and paclitaxel for the treatment of metastatic or recurrent TC. A total of 47 patients with TC were enrolled in the study, which is ongoing with promising results.

A phase IV, single-arm, multicenter study (NCT04554524) is currently underway to evaluate the first-line combination of pembrolizumab with platinum-based chemotherapy in patients with advanced TETs, including TC. This study addresses a critical therapeutic gap by assessing whether adding immunotherapy to standard chemotherapy (e.g., carboplatin and paclitaxel) improves response rates, PFS, and potentially overall survival. The trial, which enrolls patients without prior systemic therapy, uses ORR as the primary endpoint and includes secondary endpoints like safety, DCR, and duration of response to provide a comprehensive picture of efficacy.

The design of this trial holds significant potential clinical impact. By integrating ICIs earlier in the treatment course, the study aims to enhance therapeutic outcomes, particularly in patients with high PD-L1 expression or chemotherapy-sensitive histology. Additionally, biomarker analysis within this trial may inform the development of predictive indicators and establish pembrolizumab plus chemotherapy as a new first-line standard for advanced TC.

### 6.3. Integration of ICIs with Surgical Management

Perioperative integration should be anchored to resectability and margin risk. In borderline-resectable disease, a short course of platinum-based chemotherapy plus PD-1/PD-L1 blockade may downstage nodal disease and soften desmoplastic stroma; candidates merit early cardio-oncology input and a clearly defined washout before surgery. Adjuvant immunotherapy is not routine in TC given the absence of randomized data and the non-trivial risk of immune-mediated myositis/myocarditis; if pursued, it should be restricted to trials or highly selected high-risk cases. After definitive chemoradiation of unresectable presentations, consolidation ICI remains investigational and, if attempted, should follow strict cardiac surveillance with predefined steroid–taper pathways

Although the PACIFIC trial established PD-L1 blockade with durvalumab as effective consolidation therapy following chemoradiotherapy in unresectable stage III non-small cell lung cancer (NSCLC), this strategy has not been investigated in TC [76]. Its application in this context is complicated by the distinct immunological features of thymic tumors and their higher incidence of severe immune-related adverse events, particularly myocarditis and myositis. Nevertheless, accumulating preclinical and early clinical evidence indicates potential therapeutic value, highlighting the need for prospective trials to evaluate consolidation ICIs in locally advanced, unresectable TETs. Because most ICI trials exclude active or clinically significant autoimmune disease, TC-specific evidence remains limited; prospective registries are needed to refine eligibility and predict high-grade irAEs.

### 6.4. Dual ICI Therapy

Dual immune checkpoint blockade strategies aim to enhance anti-tumor immune responses by simultaneously targeting multiple inhibitory pathways [8]. A phase II trial (NCT04925947) of the dual blockade of PD1 and CTLA-4 against pembrolizumab-treated recurrent TC is ongoing with KN046, a bispecific antibody against both targets. In this study, KN046 was evaluated for efficacy and safety in patients with advanced TC that had progressed after at least one prior checkpoint inhibitor therapy. Ten patients with TC will be enrolled in phase I of this study, with the program initiated in 2021.

### 6.5. Neo-Adjuvant Therapy with ICIs

A phase II single-center, open-label, single-arm neo-adjuvant therapy study (NCT03858582) is ongoing in patients with unresectable TETs (Masaoka stage III, IVA). Patients receive pembrolizumab 200 mg, docetaxel 75 mg/m^2^, and cisplatin 75 mg/m^2^ every 3 weeks for three cycles, followed by surgical reassessment. Postoperative treatment is determined by resection status:

R0 (negative margins)—pembrolizumab for 32 cycles.

R1 (microscopic residual)—pembrolizumab plus radiotherapy (52.8 Gy/24 fractions) for 32 cycles.

R2 (macroscopic residual)—pembrolizumab plus radiotherapy (59.4 Gy/27 fractions) for 32 cycles.

The primary endpoint is major pathological response, defined as ≤10% residual viable tumor cells. This study evaluates whether immune priming with neo-adjuvant ICI–ICI-chemotherapy can improve operability and long-term tumor control in borderline resectable or locally advanced TETs. Its design integrates immunotherapy not only as a systemic therapy but also in combination with surgery and stage-adapted radiotherapy. If successful, it could inform risk-adapted multimodal treatment strategies for locally advanced thymic tumors.

### 6.6. Future Combination Therapy

The future of ICI therapy for TC also includes the development of novel immunotherapeutic agents. Bintrafusp alfa, a bifunctional fusion protein targeting PD-L1 and TGF-β [77], is currently being studied in patients with TET [78]. This innovative approach aims to enhance anti-tumor immunity by simultaneously blocking two key immunosuppressive pathways. Other emerging immunomodulatory agents, such as epacadostat (an IDO1 inhibitor) in combination with pembrolizumab, are also being evaluated for their potential to improve the prognosis of patients with TC (NCT02364076).

## 7. Conclusions and Clinical Perspectives

TC is a rare but very aggressive form of cancer with limited treatment options beyond platinum-based chemotherapy. PD-1/PD-L1–targeting ICIs show variable efficacy, benefiting some patients with high PD-L1 expression, abundant TILs, or specific genetic alterations, but still require close monitoring for immune-related adverse events. Making progress in the future will depend on consistent methods, selecting patients for treatment based on biomarkers, and developing smart combination approaches that pair ICIs with new targets (such as CD70, TIM-3, B7-H4, Trop-2, and MUC1).

## Figures and Tables

**Table 3:** Grade 3–4 irAEs in thymic carcinoma across ICI trials.

irAEs (n, %)	Pembrolizumab	Pembrolizumab	Nivolumab	Atezolizumab
	Giaccone et al. (n = 40) [10]	Cho et al. (n = 26) [11]	Katsuya et al. (n = 15) [39]	Lu et al. (n = 34) [29]
Hepatic (AST/ALT ↑)	7 (17.5%)	2 (7.7%)	3 (8%)	1 (6.7%)
Myositis/CPK ↑	4 (11%)	0	2 (5%)	0
Myocarditis	2 (5%)	0	2 (5%)	0
Myasthenia gravis/Myoclonus	0	3 (11.5%)	0	0
Other irAEs (e.g., thyroiditis, rash)	6 (15%)	1 (3.8%)	2 (5%)	2 (13.3%)
Total Grade 3–4 irAEs	~15%	~12%	~13%	~13%

irAE, immune-related adverse effect; n, number; AST, aspartate aminotransferase; ALT, alanine aminotransferase; CPK, creatine phosphate kinase. The symbol ↑ represents an increase trend.

**Table 4:** Ongoing clinical trials evaluating combination therapies in TC.

Experiment Drug	Trial	Target	Phase	End Points
Bintrafusp Alfa (M7824)	NCT04417660	PD-L1 and TGF-β	2	ORR
Pembrolizumab and Sunitinib	NCT03463460	PD-L1, PDGFR, RTK and VEGFR	2	PR and CR
Pembrolizumab and chemotherapy	NCT04554524	PD-L1	4	ORR
Pembrolizumab and chemotherapy	NCT03858582	PD-L1	2	Major pathologic response rate
Pembrolizumab, Lenvatinib and chemotherapy	NCT05832827	PD-1 and VEGFR/FGFR	2	RR
RXC004 and Nivolumab	NCT03447470	Wnt and PD-1	1	DLT
Toripalimab and chemotherapy	NCT04667793	PD-1	2	Safety and MPR
Vorolanib and nivolumab	NCT03583086	PD-1 and VEGFR/PDGFR	1/2	Escalation and Dose Expansion
KN046	NCT04925947	PD-1 and CTLA-4	4	ORR
Pembrolizumab and Lenvatinib	NCT04710628	PD-1 and VEGFR/FGFR	2	PFS

ORR, Overall Response Rate; PR, Partial response; CR, Complete response; DLT, Dose-Limiting Toxicities; MPR, major pathologic response; PFS, Progression-Free Survival.

## Abbreviations

AD	Autoimmune Disease
ANA	Antinuclear Antibody
CR	Complete Response
CTLA-4	Cytotoxic T-Lymphocyte-Associated Protein 4
DCR	Disease Control Rate
DLT	Dose-Limiting Toxicity
EMT	Epithelial–Mesenchymal Transition
ICIs	Immune Checkpoint Inhibitors
irAEs	Immune-related Adverse Events
mOS	Median Overall Survival
mPFS	Median Progression-Free Survival
MSI	Microsatellite Instability
MSS	Microsatellite Stable
MPR	Major Pathologic Response
NPR	Non-Progression Rate
ORR	Objective Response Rate
PD-1	Programmed Cell Death Protein 1
PD-L1	Programmed Cell Death Protein Ligand 1
PFS	Progression-Free Survival
PR	Partial Response
TC	Thymic Carcinoma
TET	Thymic Epithelial Tumor
TILs	Tumor-Infiltrating Lymphocytes
TMB	Tumor Mutational Burden
VEGFR	Vascular Endothelial Growth Factor Receptor
